# Selected Fungicides as Potential EDC Estrogenic Micropollutants in the Environment

**DOI:** 10.3390/molecules28217437

**Published:** 2023-11-05

**Authors:** Agata Jabłońska-Trypuć, Urszula Wydro, Elżbieta Wołejko, Marcin Makuła, Rafał Krętowski, Monika Naumowicz, Gabriela Sokołowska, Lluis Serra-Majem, Marzanna Cechowska-Pasko, Bożena Łozowicka, Piotr Kaczyński, Józefa Wiater

**Affiliations:** 1 Department of Chemistry, Biology and Biotechnology, Faculty of Civil Engineering and Environmental Sciences, Bialystok University of Technology, Wiejska 45E Street, 15-351 Białystok, Poland; u.wydro@pb.edu.pl (U.W.); e.wolejko@pb.edu.pl (E.W.); groszczenko@gmail.com (G.S.); 2 Faculty of Medical Sciences in Zabrze, Medical University of Silesia, Traugutta sq.2, 41-800 Zabrze, Poland; s68987@365.sum.edu.pl; 3 Department of Pharmaceutical Biochemistry, Medical University of Bialystok, Mickiewicza 2A Street, 15-222 Bialystok, Poland; r.kretowski@umb.edu.pl (R.K.); mapasko@gmail.com (M.C.-P.); 4 Department of Physical Chemistry, Faculty of Chemistry, University of Bialystok, Ciolkowskiego 1K Street, 15-245 Bialystok, Poland; monikan@uwb.edu.pl; 5 Research Institute of Biomedical and Health Sciences, University of Las Palmas de Gran Canaria, 35001 Las Palmas de Gran Canaria, Spain; lluis.serra@ulpgc.es; 6 Institute of Plant Protection—National Research Institute, Chełmońskiego 22 Street, 15-195 Białystok, Poland; b.lozowicka@iorpib.poznan.pl (B.Ł.); p.kaczynski@iorpib.poznan.pl (P.K.); 7 Department of Agri-Food Engineering and Environmental Management, Faculty of Civil Engineering and Environmental Sciences, Bialystok University of Technology, Wiejska 45E Street, 15-351 Białystok, Poland; j.wiater@pb.edu.pl

**Keywords:** EDC compounds, boscalid, cyprodinil, iprodione, breast cancer

## Abstract

An increasing level of pesticide exposition is being observed as a result of the consumption of large amounts of fruits, vegetables and grain products, which are key components of the vegetarian diet. Fungicides have been classified as endocrine-disrupting compounds, but their mechanisms of action have not yet been clarified. The effect of boscalid (B), cyprodinil (C) and iprodione (I) combined with Tamoxifen (T) and 17β-estradiol (E2) on cell viability, cell proliferation, reporter gene expression, ROS content, the cell membrane’s function, cell morphology and antioxidant enzymes gene expression in MCF-7 and T47D-KBluc cell lines were investigated. The cell lines were chosen due to their response to 17β -estradiol. The selected fungicides are commonly used in Poland to protect crops against fungi. Our results revealed that the studied fungicides caused significant increases in cell viability and proliferation, and estrogenic activity was present in all studied compounds depending on their concentrations. Oxidative stress activated uncontrolled cancer cell proliferation by inducing ROS production and by inhibiting antioxidant defense. Our findings verify that the studied fungicides could possibly exhibit endocrine-disrupting properties and exposure should be avoided.

## 1. Introduction

About 3 billion kg of pesticides worth approximately USD 40 billion are annually used worldwide [1]. As a result, many plant protection agents known and described as endocrine-disrupting compounds that affect fertility are constantly present in the environment and in humans and animals [2]. The human population is most often exposed to mixtures of different types of pesticides present in air, water and food [3]. The research results available in the literature do not clearly define fungicides’ effects on the human body. Due to the widespread use of fungicides in agriculture, both active compounds, their metabolites and their residues are often found in plant-derived food products obtained from various crops. The consumption of fruits, vegetables and grain products causes people to be increasingly exposed to active substances of pesticides. In addition to absorption in the digestive system, these compounds can enter the human body through the respiratory tract and skin. The last two routes of fungicide penetration into the human body are particularly important in relation to employees working directly with fungicides, e.g., people who mix compounds, fill spraying equipment and spray crops. Based on our own research, it was found that herbicides, which are also described as plant protection products safe for humans, show both carcinogenic and estrogenic potential [4]. The results above constitute the basis for the design of our experiment aimed at studying the potentially estrogenic effects of selected fungicides.

Among the fungicides, the most commonly used originate from the group of succinate dehydrogenase inhibitors (SDHIs). Their mechanism of action is the inhibition of succinate dehydrogenase (SDH) complex II of the mitochondrial electron transport chain (ETC) [5]. Boscalid (B) is one of the most popular compounds from this group. Generally, SDHIs are considered safe, but recent literature data proved that SDHIs applied in order to obtain fungicidal effects did not only show activity against target organisms but also blocked SDH in honeybees, earthworms and humans. It may be due to the fact that there is a high degree of conservation of a large proportion of the SDH sequence present in a variety of organisms. Even partial SDH malfunctions have also been shown to increase susceptibility to SDHI [6,7]. Boscalid is characterized by good solubility in water, which is why it is found in the aquatic environment (Table 1). The highest concentrations of boscalid identified in surface and groundwater were 2120 μg/L and 0.109 μg/L (United States), respectively. Due to its effect on the electron transport chain in mitochondria, this compound is also active against non-target organisms, such as the algae Chlorella vulgaris and zebrafish. The results of studies conducted using zebrafish indicate an obvious increase in the level of oxidative stress and cytotoxicity and apoptosis parameters after exposure to boscalid [8,9,10]. The literature data indicate an evident effect of boscalid on the formation of neurodevelopmental defects in zebrafish models, changes in main macromolecule metabolism, kidney and liver malfunctioning, and genotoxic effects in various types of human cells [11,12,13,14]. It turns out that even a short incubation in the presence of boscalid initiates the processes of mitochondrial damage in human cells of the following lines: Peripheral Blood Mononuclear Cells or PBMCs, HepG2 liver cells, and BJ-fibroblasts [15].

Cyprodinil (C), on the other hand, is a frequently used fungicide from the group of pyrimidinamine compounds [17]. It is mainly used in agriculture for the protection of orchards, vines, cereals and vegetables against various pathogens. Cyprodinil is also commonly identified in the aquatic environment. Its concentration range is between 0.736 and 2.898 μg/L in the aquatic environment (Chile). Due to the fact that it is easily metabolized in the environment, many of its metabolites appear with unknown structures and potential effects on living organisms (Table 1). [18]. The literature data indicate that cyprodinil causes phosphorylation of extracellular signal-regulated kinase (ERK), which phosphorylates growth factors and transcription factors. ERK regulates differentiation, migration, proliferation and survival and activates ER signaling. Moreover, cyprodinil, as an activator of the aryl hydrocarbon receptor (AhR), induces cytochrome P450 in ovarian granulosa cells at the gene level. It potentially affects reproductive function by activating both AhR and ERK signaling. Moreover, the carcinogenic potential of cyprodinil was demonstrated as a result of studies on the induction of ovarian cancer proliferation through the ER-dependent pathway. Therefore, it can be argued that cyprodinil may act as a cellular disruptor of human physiology [19,20,21]. The biological effect of cyprodinil at the cellular level consists in inhibiting the synthesis of amino acids, including methionine and other thionic amino acids, and interfering with fungal metabolism [22,23]. In cell lines representing CNS glial and neuronal cells such as U251 and SH-SY5Y, cyprodinil was the most toxic agent among the fungicides tested, although an increase in the gene expression of the oxidative stress enzyme shows a certain level of synergism in the presence of a mixture of active substances such as pyrimethanil and fludioxonil [24].

Iprodione (I) belongs to the group of dicarboxamide fungicides applied to prevent and treat sclerotonia and gray mold in vegetables and fruits [25]. Iprodione has been classified in Group B2 as a “probable” human carcinogen (Table 1). This classification is based on evidence from studies on the induction of liver tumors in mice and rats. Studies indicate that exposure of mice to iprodione changes the activity of microsomal enzymes, increases hepatocyte proliferation and may cause hepatomegaly. Moreover, as an endocrine active substance, it causes reproductive disorders in rats. However, data on the cytotoxicity, genotoxicity and potential carcinogenicity of iprodione are very poor. Therefore, the OECD commission introduced regulations related to the lack of approval for the introduction of iprodione for use [26,27,28,29].

Research results indicate that not only the active parent substance of the pesticide but also iprodione metabolites pose a threat to the natural environment and the organisms living in it [30]. Failure to assess the toxicity risk of metabolites usually leads to an insufficient assessment of the safety of pesticides themselves. In the environment, pesticides basically coexist with their metabolites; therefore, assessing the ecological safety of their metabolites is necessary for a comprehensive understanding of the negative impact of pesticides on the environment and human health [31]. There are also reports that iprodione is not only effective against fungal pathogens, but it is also toxic to non-targeted, aquatic organisms [32]. The US Environmental Protection Agency (EPA, Washington, DC, USA) has reported that 3,5-DCA (3,5-dichloroaniline), the major metabolite of iprodione, is classified as a carcinogen that is characterized by a higher level of toxicity and persistency than the parent compounds [33]. Although the available data on the toxicity of iprodione are limited, it is known that it has an anti-androgenic effect manifested by reducing testosterone levels and delaying the sexual development of male rats [34]. Although the literature data indicate that iprodione modifies the synthesis of estrogens and androgens, the mechanisms underlying iprodione-induced reproductive toxicity are not yet known and described [35]. Low doses of iprodione residues, often found in various fruits and vegetables, affect the functioning of the human hormonal system because of its endocrine-disrupting activity [36,37]. In addition, iprodione residues have been identified in products such as garlic, green leafy vegetables, grapes and tropical crops, where its use is not legally allowed [38]. The literature data indicate that iprodione may influence the timing of sexual maturation and change the metabolism of steroids in the testes of male rats by inhibiting their synthesis [34]. Fungicides such as iprodione can inhibit sterol biosynthesis, energy production, amino acid synthesis and cell division. Therefore, these compounds targeted against these processes or functional groups may be, to some extent, toxic to the body. Iprodione is a phytotoxic and cytotoxic compound that causes chromosomal changes and cell death [39]. Considering the fact that, when comparing with other fungicides, this compound has a very long half-life, its mechanisms of action should be carefully examined [40].

The main goal of the present study was the evaluation of the potential estrogenic properties of the active substances of selected fungicides, boscalid, cyprodinil and iprodione, by using the following in vitro bioassays for measuring estrogenic activity: estrogen-dependent (ER+) human breast cancer cell line (MCF-7) proliferation test or E-screen test and luciferase transfected human breast cancer cell line T47D-KBluc gene-reporter assay. Cells were selected for their response to 17-beta-estradiol (E2). They can be used to test chemicals for their estrogenic or anti-estrogenic activity. A further aim of this study was to analyze oxidative stress parameters, membrane functioning and cell morphology under the influence of fungicides and compare these parameters with proliferation test results in order to establish possible relationships between the phenomenon of oxidative stress and cancer development stages. The selected active substances of fungicides were chosen because they are agents commonly used in Poland to protect crops against fungi.

## 2. Results

### 2.1. Cytotoxicity

**Figure 1:**
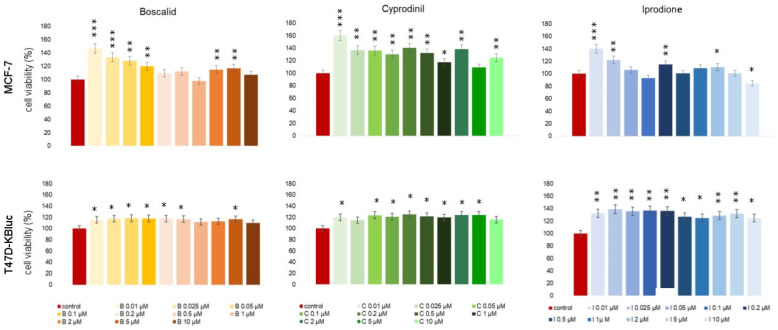
Viability of MCF-7 and T47D-KBluc cells exposed to boscalid, cyprodinil and iprodione for 24 h (percentage of untreated cells). Each value on the graph is the mean of three independent experiments, and error bars show the standard deviation (SD). * *p* < 0.05, ** *p* < 0.01, and *** *p* < 0.001 represent significant effects between treatments and controls followed by a Dunnett’s test.

In order to estimate the potential cytotoxicity of the test compounds, an MTT assay was applied (Figure 1.). It allows us to determine the viability of cells exposed to the tested fungicides. In the MCF-7 cell line, an increase in the studied parameter was noticed in the first two analyzed concentrations—0.01 µM and 0.025 µM. In the case of boscalid, the increase was almost 47% relative to the control, for cyprodinil—about 60%, and for iprodione—about 40% relative to untreated control cells. None of the tested compounds decreased cell proliferation in a statistically significant manner when compared with non-treated cells. In T47D-KBluc cells, an increase in cell viability was also noticed as a result of the tested fungicides exposition; although, regardless of the concentration of compounds, increases were at similar levels. The mean increase in proliferation observed in the T47D-KBluc cell line was approximately 15% for boscalid, 21% for cyprodinil and 31% for iprodione. Observed changes in cell viability were statistically significant for all compounds tested.

**Figure 2:**
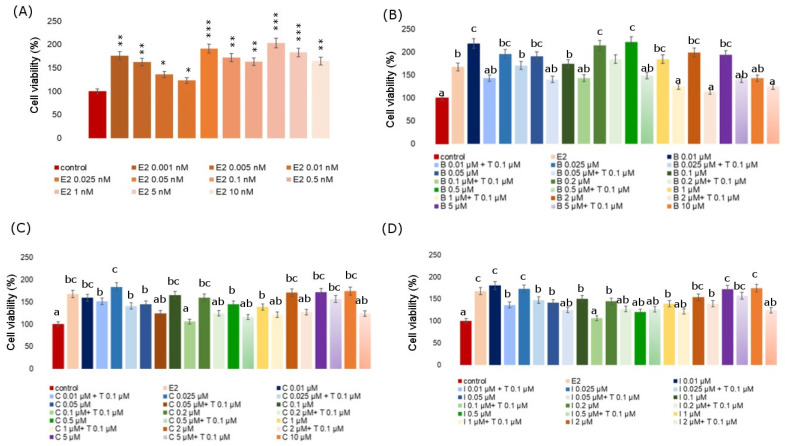
Viability of MCF-7 cells incubated with different concentrations of E2 (**A**), different concentrations of B combined with 0.1 µM Tamoxifen (**B**), different concentrations of C combined with 0.1 µM Tamoxifen (**C**), different concentrations of I combined with 0.1 µM Tamoxifen (**D**). Mean values from three independent experiments ± SD are presented. Significant alterations are expressed relative to control cells (marked with asterisks). Statistical significance was at * *p* < 0.05, ** *p* < 0.01, *** *p* < 0.001. Different letters indicate statistical differences (*p* ≤ 0.05) between treatments estimated by Tukey’s test.

An MTT test was also carried out on the MCF-7 cell line with the use of a positive control, i.e., 17β-estradiol and the simultaneous application of Tamoxifen at a concentration of 100 nM (Figure 2). Tamoxifen is a nuclear estrogen receptor blocker in this study. MTT for estradiol was performed over a concentration range from 0.001 nM to 10 nM. At all analyzed concentrations, estradiol caused an increase in the viability of MCF-7 cells, an especially strong effect was noticed for the 1nM concentration, where this increase exceeded by more than 100% of the control. The average increase in the level of viability of cells exposed to estradiol was over 67% in reference to the data for the control. Subsequently, the MCF-7 cell line was treated with a fungicide and with fungicide and Tamoxifen simultaneously. In each analyzed case, there was a significant increase in proliferation as compared to the negative control under the influence of fungicide and Tamoxifen. In the cases of boscalid and iprodione, the first analyzed concentration of 0.01 µM resulted in a significant increase in cell viability, even as compared to the estradiol-treated culture. However, the addition of Tamoxifen significantly decreased cell proliferation when compared to the untreated cells and compared to boscalid alone. A similar situation was observed for the iprodione. For cyprodinil, the second of the analyzed concentrations—0.025 µM—caused the highest significant increase in cell proliferation with an increase of over 80%, and the simultaneous administration of 0.1 µM Tamoxifen decreased the tested parameter by over 40%.

### 2.2. E-screen Assay and T47D-KBluc Gene-Reporter Assay

**Figure 3:**
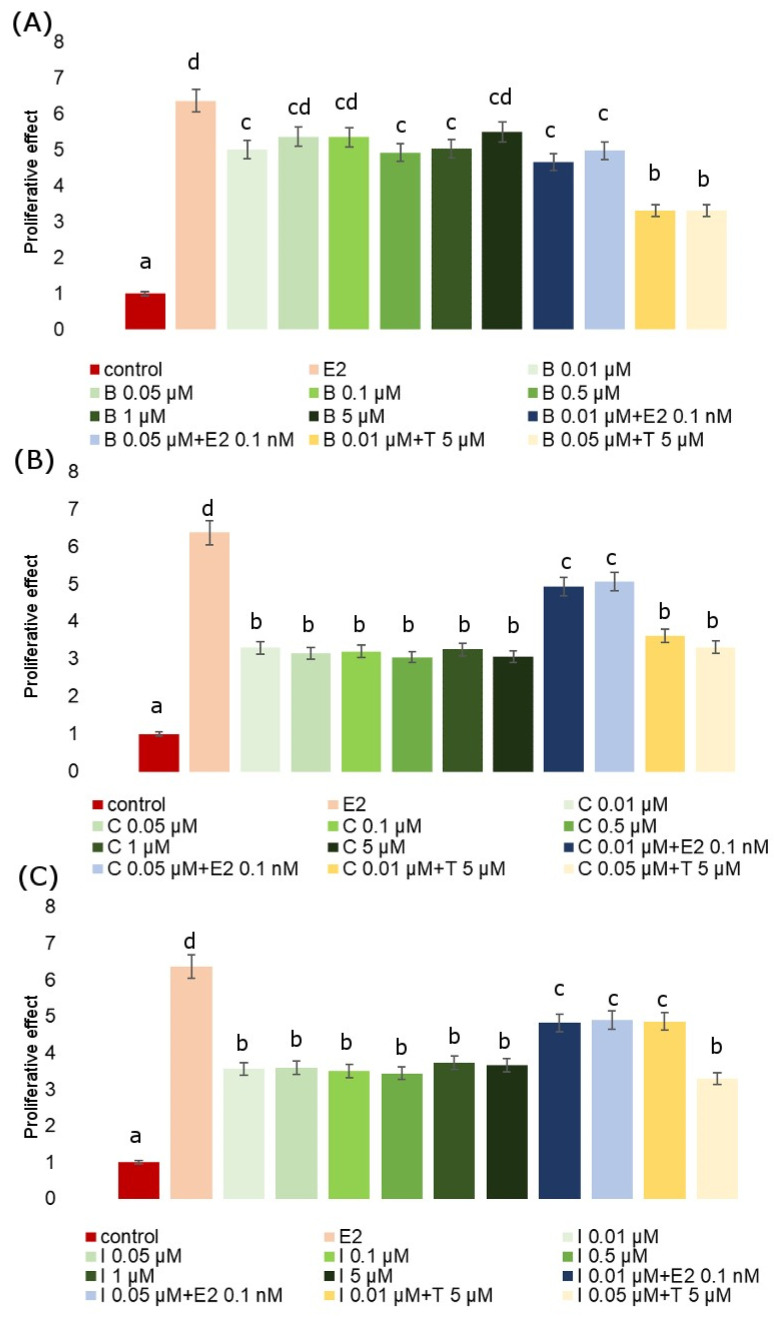
E-screen assay. Maximum proliferative effects (PE) induced by B (**A**), C (**B**) and I (**C**) in MCF-7 cells. PE mean values of fungicide co-incubated with Tamoxifen (5 µM) and with E2 (0.1 nM). The values represent the means ± standard deviations. Different letters indicate statistical differences (*p* ≤ 0.05) between treatments estimated by Tukey’s test.

The E-screen assay demonstrates a potential increase in the proliferation of human breast cancer cells (MCF-7) after exposition to estrogenic substances. It serves for the rapid assessment of the estrogenic activity of potential xenoestrogens (single compounds and mixtures). It is a type of bioassay that allows for the measurement of the increase in the number of human breast cancer cells (MCF-7 cell line) exposed to estrogen. Exposition to all analyzed fungicides resulted in an increase in MCF-7 proliferation when compared to untreated cells. The proliferation-stimulating effect of fungicides in MCF-7 cells with respect to the positive control (estradiol) is presented in relation to RPE% in Table 2. The RPE of the fungicides generally exhibited partial agonistic action (RPE < 100). The proliferative effect (PE) of the studied fungicides and their combinations with Tamoxifen and estradiol is presented in Figure 3. We observed that co-incubation with Tamoxifen evidently inhibited cell proliferation, whereas co-incubation with estradiol increased the proliferative response, demonstrating that the observed cell proliferation was ER-dependent. In every analyzed case we observed significant effects. The mean value of the viability index of exposed to estradiol MCF-7 cells showed in the E-screen test was higher when compared with the negative control. Also, the viability of cells exposed to all tested fungicides was significantly higher compared to the control, but for each fungicide, it was lower than for estradiol. Co-incubation of MCF-7 cells with an estradiol-combined fungicide increased cell proliferation, which was particularly evident for iprodione and cyprodinil.

The results obtained as a result of the test of the estrogen receptor reporter gene on the T47D-KBluc cells confirm those obtained in the E-screen test, indicating the estrogenic properties of the tested compounds. TRANS% values are the highest at the highest concentrations of fungicides tested, which may indicate that these compounds are dangerous to the human endocrine system in a wide range of concentrations. However, it is worth noting that even the lowest concentrations of fungicides show relatively high TRANS% values, indicating fungicides as EDC compounds.

### 2.3. Oxidative Stress

**Figure 4:**
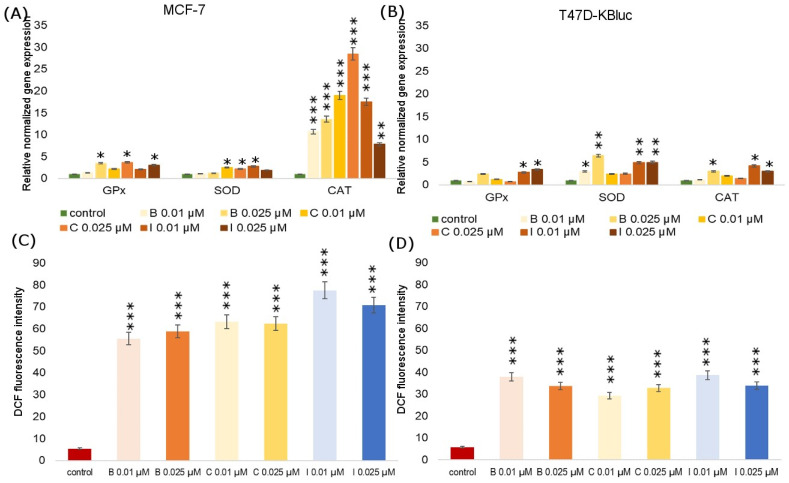
The influence of boscalid, cyprodinil and iprodione on GPx (glutathione peroxidase), SOD (superoxide dismutase) and CAT (catalase) relative gene expression in MCF-7 (**A**) and T47D-KBluc (**B**) cells. The effect of boscalid, cyprodinil and iprodione on the level of intracellular ROS in MCF-7 (**C**) and T47D-KBluc (**D**) cells. The cells were incubated with B (0.01 µM, 0.025 µM), C (0.01 µM, 0.025 µM) and I (0.01 µM, 0.025 µM) for 24 h. Mean values from three independent experiments ± SD are shown. Significant alterations are expressed relative to control cells (marked with asterisks). Statistical significance was at * *p* < 0.05, ** *p* < 0.01, *** *p* < 0.001.

Tested compounds showed a high carcinogenic potential, which was manifested, inter alia, by the observed changes in oxidative stress parameters. The influence of B, C and I on the expression of genes encoding antioxidant enzymes (catalase, glutathione peroxidase, superoxide dismutase) was investigated in MCF-7 and T47D-KBluc cells (Figure 4). Particularly significant increases in the genes encoding all studied enzyme expressions were noted in the T47D-KBluc line, especially in the case of SOD. On the other hand, in the MCF-7 line, an increase in the CAT coding gene expression was observed under the influence of all analyzed fungicides at all tested concentrations. There were only two cases where gene expression fell below the negative control level. In the T47D-KBluc line, under the influence of B at a concentration of 0.01 µM and under the influence of C at a concentration of 0.025 µM, the expression of the GPX gene decreased.

Figure 4 presents the effects of fungicides on ROS production in MCF-7 and T47D-KBluc cells. The relative amount of ROS is shown as the DCF fluorescence intensity in the cells of both tested cell lines cultured with B, C and I, each at two concentrations of 0.01 µM and 0.025µM. Incubation of cells with pesticides increased the content of ROS significantly over 10 times compared to the control. No decrease in the ROS level below the negative control was observed in any of the tested lines and under the influence of any of the tested fungicides. The obtained results indicate the strengthening effect of pesticides on the formation of ROS, which at the same time translates into the stimulation of proliferation and tumor growth.

### 2.4. Effects of the Fungicides on the Zeta Potential and Surface Charge of Cell Membranes

**Figure 5:**
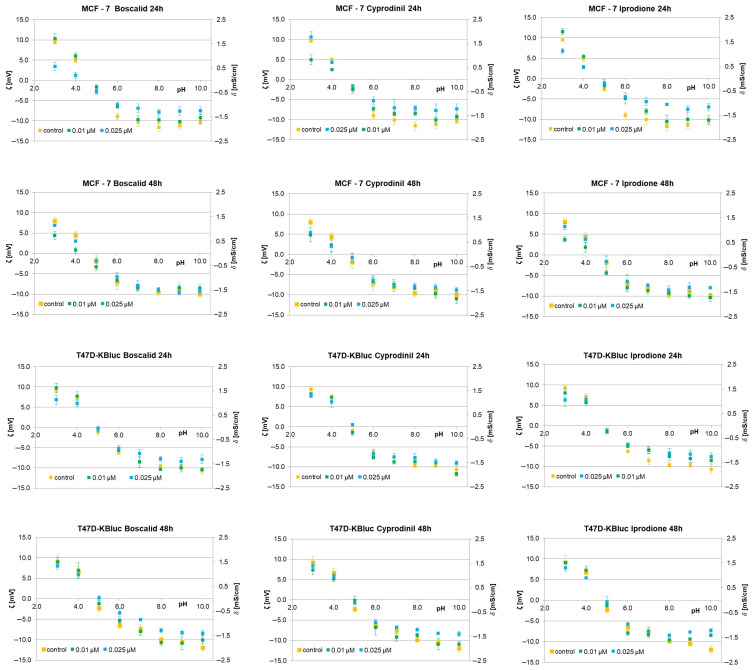
The zeta potential (left axis) and the corresponding surface charge (right axis) of MCF-7 and T47TD-KBluc cells versus pH of electrolyte solution. The cells were untreated (orange) or treated with 0.01 μM of fungicide (green) and 0.025 μM (navy blue) of fungicide for 24 h and 48 h.

Zeta potential (*ζ*) and surface charge (*δ*) are physicochemical parameters that are related to the cell membrane composition and play an essential function in the interaction of cells with ions, molecules and surfaces. Both of these parameters can be obtained using an electrophoretic light scattering method grounded in dynamic light scattering, which uses the phase shift of the oscillation or frequency of the laser beam to depend on the mobility of the particles/cells in an alternating electric field [41]. From the viability test results, E-screen assay and T47D-kBluc gene-reporter test, it can be seen that all the tested fungicides cause augmented proliferation of MCF-7 and T47D-kBluc cells. While various reasons may be responsible for this proliferation effect, one plausible explanation may be related to the specific way in which compounds interact with the cell membrane. To verify if fungicides interact with/adsorb on the cell membrane, microelectrophoretic mobility determinations were carried out for MCF-7 and T47D-KBluc (Figure 5) cells treated with boscalid, cyprodinil or iprodione for 24 h and 48 h. Measurements were carried out at pH ranging from 2 to 10, with NaCl as a supporting electrolyte. Values *ζ* were obtained from Equation (1), and *δ* values were calculated using Equation (2). Mean values with standard deviations were given for quantitative data. The *ζ* and *δ* dependencies of MCF-7 and T47D-KBluc cells on pH gave the same shaped curves for all determinations: an increase in positive values of both electrical parameters of the cells at low pH values and in their negative values at high pH values until a plateau was obtained.

The surface charge and zeta potential values versus pH for MCF-7 cell membranes are plotted in Figure 5. The cells were cultured with 0.01 and 0.025 mmol/dm^3^ of boscalid, cyprodinil or iprodione for 24 h and 48 h. As noted, all three fungicides affected the values of the evaluated parameters. The contents of these compounds caused a decrease in positive values of the *ζ* and *δ* at acidic pH levels. On the other hand, the negative values of both parameters decreased at neutral and alkaline pH (at pH greater than the pH of the isoelectric point). These alterations seemed to be dose- and time-dependent as they are much more pronounced with both higher fungicide concentrations and shorter incubation times. Moreover, no notable alterations were noticed in the isoelectric point values of the MCF-7 cells treated with boscalid, cyprodinil or iprodione (irrespective of dose and treatment time).

The T47D-KBluc cells treated with the analyzed fungicides were examined analogously. Figure 5 shows representative plots of both zeta potential and surface charge vs pH obtained for T47D-KBluc cell membranes cultured with boscalid, cyprodinil or iprodione for 24 h and 48 h. It is evident that replacing MCF-7 cells with T47D-KBluc cells results in less pronounced changes in the *δ* and *ζ* values obtained for fungicide-treated cell membranes compared to control membranes.

Based on the data shown in Figure 5, one might come to the conclusion that the studied fungicides slightly modulated the electrical parameters of MCF-7 and T47D-KBluc cell membrane surfaces, although no difference between boscalid, cyprodinil and iprodione was sufficiently marked to definitively conclude a stronger charge-modulating influence of the tested compounds. The changes in the electrical parameter values were less pronounced when the cells were incubated with fungicides for 48 h compared to the changes obtained within 24 h of incubation, suggesting that the latter were mainly present inside the cells (rather than on their surfaces).

### 2.5. LC–ESI–MS/MS Analysis of Boscalid, Cyprodinil and Iprodione in MCF-7 Cells

As is indicated in Table 3, which presents the amount of each fungicide that entered the cells, statistically significant amounts of compounds entered the cells. In the case of boscalid at 0.01 µM concentration, more than 70% of the compound entered cells, and more than 77% of cyprodinil at the same concentration entered cells. It is evident that the tested pesticides penetrate the cells in greater amounts when they are applied in a lower concentration. This confirms the results of the cell proliferation assays, which show the highest increase in proliferation at the lowest concentration tested. This is true for all those studied in the MCF-7 cell line fungicides.

### 2.6. Morphological Changes in Live Cells

**Figure 6:**
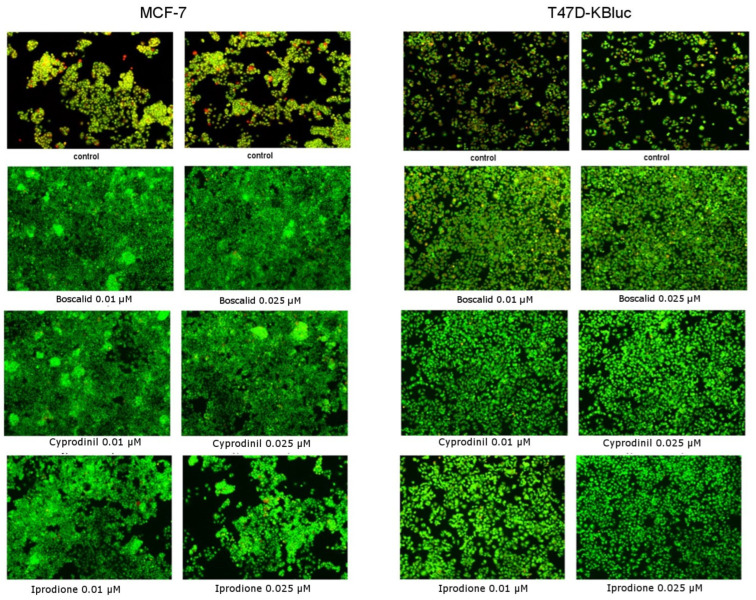
The influence of boscalid, cyprodinil and iprodione on apoptosis/necrosis in the MCF-7 and in the T47D-KBluc cell lines studied with the use of fluorescence microscope (200× magnification). The cells were exposed to B (0.01 µM, 0.025 µM), C (0.01 µM, 0.025 µM) and I (0.01 µM, 0.025 µM) for 24 h and stained with acridine orange and ethidium bromide (red color—apoptotic cells, green color–live cells). Representative images from one of three independent experiments are presented.

**Figure 7:**
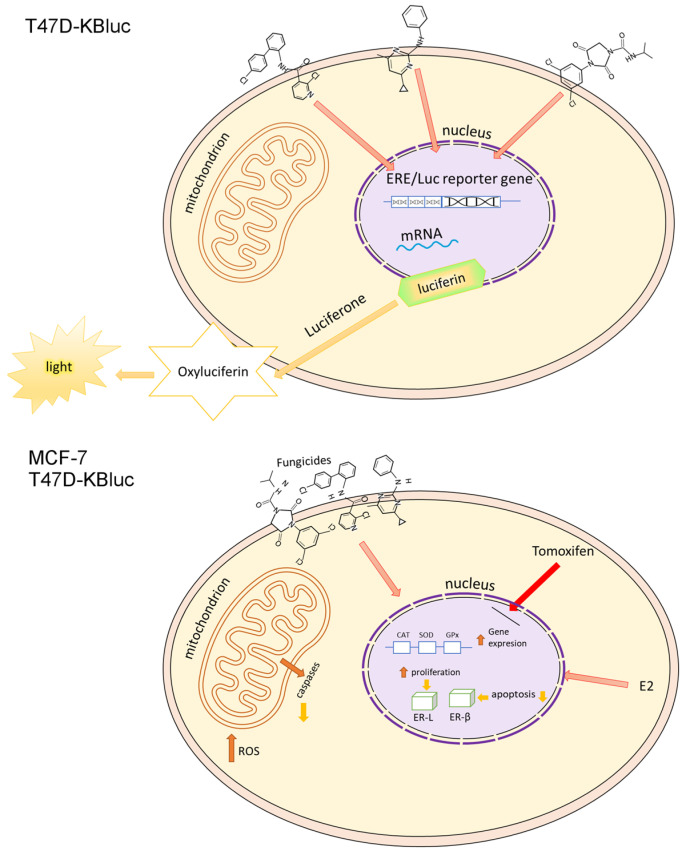
Tentative model of fungicides’ mechanism of action in T47D-KBluc and MCF-7 cells.

Cell staining with acridine orange and ethidium bromide was applied in order to evaluate morphological changes in live cells (apoptotic/necrotic). In living cells, cell nuclei stained green with a simultaneous lack of chromatin condensation were observed. Apoptotic cells were characterized by visible chromatin condensation and red or green staining of the nuclei. On the other hand, in necrotic cells, red staining of the nuclei was demonstrated but without visible chromatin condensation. In Figure 6 and Figure 7, it was shown that the fungicides tested did not reduce the viability of the cells and even stimulated their proliferation. There were no significant changes in the morphology of both cells and cell nuclei and no differences between cells incubated with 0.01 µM or 0.025 µM concentrations of compounds. In contrast, in the case of control cells, apoptotic cells characterized by fragmented and marginalized chromatin were observed, with shrinkage of the nuclei compared to the treated fungicides. No significant changes in cell proliferation rates were noticed, which confirms the MTT test results mentioned earlier.

## 3. Discussion

The universality and inappropriate use of fungicides in plant protection are associated with the detection of an increasing amount of their residues in food products. Consumption of such products can significantly increase human exposure to fungicides and thus induce carcinogenesis processes, subsequently causing significant changes in the endocrine system. However, it should be remembered that crops left without human interference have a greater possibility for the development of fungal diseases and thus a quantitative and qualitative drop in the final yield. For this reason, the use of chemical substances that control the growth of undesirable pathogenic microorganisms has become an important and constant element of modern plant cultivation technologies. Fungicides, next to herbicides, belong to one of the most commonly used groups of plant protection products that minimize the development of fungi and thus provide optimum conditions for the development of crops. Properly used fungicides allow us to achieve high crop efficiency due to the elimination of fungal diseases without causing major damage to the natural environment. Selected fungicides in which the active substances are boscalid, cyprodinil and iprodione are now frequently found in food products. This is due to the fact that they are widely used to protect crops in Poland. The preliminary study of the biological activity of these selected fungicides on the viability of cells of two breast cancer cell lines made it possible to estimate the potentially estrogenic activity of the studied compounds, and it is an introduction to further studies of the molecular mechanisms by which selected fungicides act.

This paper focuses mainly on the study of the potentially estrogenic properties of selected fungicides, and both the E-screen test and the T47D-KBluc gene-reporter test turned out to be the right choice to prove the estrogenic properties of the tested compounds.

The studied fungicides showed estrogenic activity that was lower than that of E2 and lower than that of a mix of fungicides with E2. The obtained results show that cyprodinil and iprodione are almost twice less potent than E2, whereas boscalid is only 1.2 times lower than E2. It has previously been described in the literature that pesticides have varying levels of estrogenicity [42], and these results and the conclusions drawn from them should be considered when determining exposure levels because estrogenic action is an important factor in biological effect [43]. Our findings are in agreement with the results presented in our other, previously published works, where we observed cancer cell growth stimulation caused by different types of pesticides, mainly herbicides, which exhibit cancer-promoting activity [4,44,45]. Based on our previous experiments, we concluded that pesticides act as triggers and promoters of cancer, including estrogen-dependent cancers. That is why we decided to conduct further studies using the most popular fungicides in Poland and Europe in order to see if they act as endocrine disruptors.

First, using the MTT assay, we investigated the viability of the MCF-7 and T47D-KBluc cells under the influence of boscalid, cyprodinil and iprodione. Then, the cells of the MCF-7 line were exposed to the test compounds with estradiol for 24 h and, with the test compounds, co-incubated with 0.1 µM Tamoxifen, which blocks estrogen receptors. In the MCF-7 cells, the most significant increases in proliferation were noticed at the lowest concentration of each analyzed compound tested. It was a concentration of 0.01 µM, which is in agreement with the results of the LC–ESI–MS/MS analysis. According to the obtained results, each of the tested compounds penetrated into the cells to a greater extent at a lower concentration (0.01 µM) than at a higher concentration (0.025 µM). The decreases in cell proliferation below the control were statistically insignificant and they were observed in only two cases—1 µM boscalid and 0.1 µM iprodione. No decrease in proliferation below the control level was observed in the T47D-KBluc cell line. The available literature data report that pesticides can stimulate the proliferation of cancer cells using various mechanisms, e.g., glyphosate acts as an EDC compound stimulating the growth of breast cancer by activating estrogen receptor pathways (both ER-α and ER-β), ROS mediate in the induction of diuron toxicity, while herbicides such as bifenox and dichlobenil stimulate oxidative stress, while increasing the proliferation of cancer cells and inhibiting apoptosis [4,46,47]. An instance of inhibition of apoptosis under the influence of the analyzed fungicides was observed in our microscopic study. Our results indicating the estrogenic activity of pesticides from the fungicide group are consistent with the literature data confirming the fact that various pesticides act as EDC compounds (Figure 7). Among others, Schiliro et al. confirmed the existence of a positive correlation between the presence of pesticide residues in selected species of vegetables and fruits and their estrogenic activity [48]. The above-mentioned authors, like us, used the E-screen test to demonstrate the estrogenic activity of the tested compounds. They also used gene-reporter assay but based on a different cell line—MELN. In turn, in the work of Soto et al., one of the tests used to investigate estrogenic environmental pollutants was also the E-screen assay [49]. The compounds tested included, among others, herbicides, insecticides and fungicides.

The T47D-KBluc gene-reporter assay (Table 2) confirmed the estrogenic effect of the tested fungicides. It is generally a useful and good method of detecting estrogenic activity in various samples, including environmental ones, but it is not always sensitive enough. Therefore, we did similarly to Chou et al., and to improve the estrogen test sensitivity, we included T47D-KBluc cells grown in medium without phenol red for 12h and subsequently exposed the cells to the test substances. The literature data show that such an effect significantly stimulates the estradiol (E2)-induced activation of luciferase and thus its sensitivity and precision [50]. A similar method, also based on T47D-KBluc cells, was used by Wehmas et al. [51].

Compounds acting as endocrine disruptors may manifest their activity through a direct activating or inhibiting effect on estrogen receptors (ER) or androgen receptors (AR). They can also stimulate the aryl hydrocarbon receptor (AhR) pathway, which produces an anti-estrogenic effect without direct action on the ER receptor. The binding of chemical substances to the above-mentioned receptors proves, in a sense, their nature as endocrine disruptors [52,53]. The literature data indicate that cyprodinil is an AhR, a low ERα agonist and a weak antagonist of AR. Pesticides influence estrogen signal pathways in various ways, modulating, among others, the expression of ER-dependent genes such as NRF-1, CCND1 and PGR. ERα expression can be changed after co-incubation with E2, while ERβ increases its expression only under the influence of specific pesticides. [54]. This effect could have occurred in our studies, especially for cyprodinil. Co-treatment of the MCF-7 cell line with cyprodinil and E2 clearly increased its proliferative effect compared to cyprodinil alone. Moreover, an inhibitory effect of Tamoxifen was observed. It was similar in the case of iprodione, but the inhibitory effect of Tamoxifen was not as obvious. The observed increase in the proliferation of MCF-7 cells in lower concentrations of the tested fungicides and the decrease in the level of this parameter with the increase in the concentration of the compounds under study are consistent with the literature data. However, according to Medjakovic et al., these changes are not related to the status of AR and ER receptors and may be partially dependent on AhR because AhR is associated with the cell cycle [54].

Due to the undeniable relationship between the stimulation of oxidative stress parameters and the induction of cancer development, we also decided to investigate selected stress parameters. Substances classified as EDCs, including the fungicides we tested, undoubtedly play an important role in carcinogenesis processes. The literature data indicate that primarily estrogen-dependent cancers, where E2 enhances the growth of tumors and promotes their metastasis, and EDCs compounds enhance this cancer progression [55]. According to Kim et al., cyprodinil together with E2 promotes the migration and invasion of Ishikawa endometrial cancer cells, which are estrogen-responsive [56]. However, Go et al. confirm the ability of cyprodinil to stimulate cell cycle progression in an estrogen-dependent ovarian cancer model [19]. Considering oxidative stress parameters, cancer cells are generally characterized by increased levels of ROS in comparison to healthy cells [39]. It results from the genetic disorders that occur in them and, as a result, they cause uncontrolled proliferation. In our study, we did not notice any decreases in ROS levels in cells treated with fungicides compared to the control cells. Generating ROS to combat the body’s natural antioxidant protection may be a mechanism, in addition to acting on the estrogen receptor pathway, that stimulates tumor growth. In order to further analyze the oxidative stress generated by fungicides in MCF-7 and T47D-KBluc cells, we performed molecular verification. We examined the expression of genes encoding SOD, CAT and GPX with simultaneous exposure to boscalid, cyprodinil and iprodione. In the current study, SOD transcription was significantly altered in the T47D-KBluc cells, and thus, SOD is considered to be one of the major regulatory enzymes that induce oxidative stress. In the MCF-7 cells, a particularly significant increase in the expression of the CAT gene was noticed. The T47D-KBluc cell line was characterized by a generally higher expression of genes encoding antioxidant enzymes (Figure 7). Our results turned out to be consistent with those obtained by Aksakal F.I., where an increase in catalase activity as a result of exposure to boscalid was also noted [57]. However, we did not observe a decrease in SOD relative to normalized gene expression in any of the tested fungicides but in the case of boscalid similar to the above-mentioned author. To sum up, an increase in the level of ROS and changes in the activity of antioxidant enzymes may undoubtedly be one of the parameters contributing to the cellular redox imbalance which is found in many types of cancer cells compared to healthy ones. Such redox imbalance may be an additional factor stimulating the oncogenic activity of the selected pesticides.

The electrophoretic light scattering technique has already been applied in our previous works to study the surface charge and/or zeta potential of human glioblastoma cells treated with phenolic compounds [58] and the cell membranes of red blood cells and platelets exposed to polymer particles [59]. Based on the data shown in Figure 5, one might come to the conclusion that the studied fungicides slightly modulated the zeta potential and surface charge values of MCF-7 and T47D-KBluc cell membrane surfaces, although no significant difference between boscalid, cyprodinil and iprodione was noticed to definitively state a stronger charge-modulatory effect of any of these compounds. Changes noticed in the values of the electrical parameters were less pronounced after a longer incubation time (48 h) of the cells with the fungicides as compared to the changes obtained after a shorter incubation time (24 h), suggesting that the fungicides were mainly present inside the cells (rather than on their surfaces). In addition to the above-mentioned parameters, other physicochemical properties may also play a role in a fungicide’s interaction with cell membranes and its ability to permeate them, such as the partition coefficient in an *n*-octanol/water system (log P_OW_), topological polar surface area (TPSA) or rotatable bound count. Log P_OW_ is a common assay used to measure the hydrophilicity or lipophilicity of a substance. An extensive review of drug absorption demonstrated the usefulness of log Pow in the assessment of a compound’s permeation and absorption. One average value of optimum log Pow was 2.94 [60]. Regarding boscalid, cyprodinil and iprodione, the reported log Pow values are 2.96, 3.59 and 3.0, respectively [61]. Thus, the fungicides under analysis in the present research have a higher affinity to the lipophilic phase (cell membranes) than to the aqueous phase (culture media or electrolyte solution). The rotatable bond count helps to predict passive membrane permeation across the membrane; a count of less than 10 rotatable bounds is an indicator of potentially higher permeation [62]. All our compounds fall within this range, the rotatable bonds count being three for boscalid and cyprodinil and two for iprodione [61]. The polar surface area represents the sum of oxygen, nitrogen and bonded hydrogen atoms, which are the main functional groups present on the cell surface. To facilitate the use of this parameter, more recent studies use the TPSA, proposing that molecules with TPSA > 140 Å^2^ show low membrane permeability, while compounds with TPSA < 60 Å^2^ have a higher ability to penetrate biological membranes [45]. Iprodione presents the highest value (69.7 Å^2^), while boscalid (42 Å^2^) and cyprodinil (37.8 Å^2^) present progressively lower TPSA values [61]. Combining zeta potential and surface charge values with log Pow, rotatable bound count and TPSA values, we can assume that the studied fungicides can penetrate cell membranes. It is in agreement with the LC–ESI–MS/MS analysis results indicating rather easy and efficient penetrations of boscalid, cyprodinil and iprodione into the cells. An observer interaction of fungicides with the cell membranes could be a possible explanation for the induction of oxidative stress pathways and, at the same time, estrogenic receptor interactions.

The aim of our research was to obtain a more detailed insight into the mechanisms of selected fungicides in estrogen-dependent cancer cells. Various parameters were examined, both related to oxidative stress and the endocrine-active properties of the tested compounds. Such extensive research, including the identification of pesticides in cells and determining their ability to penetrate cell membranes and interact with these membranes, allowed us to draw conclusions. We conclude that the tested fungicides may pose a certain risk to human health because they clearly change molecular parameters important from the point of view of proper physiology. They can be classified as EDCs, which is quite a serious danger and imposes the need for, and even necessity for, further research to explain the molecular paths of their action at the cellular level, as well as at the level of the entire human organism.

## 4. Materials and Methods

### 4.1. Chemical Treatment of Cells

The human cell lines MCF-7 and T47D-KBluc were obtained from American Type Culture Collection (ATCC, Manassas, VA, USA). The MCF-7 cells were grown in EMEM supplemented with 10% FBS (Gibco) at 37 °C in a humified atmosphere of 5% CO_2_ in air. The T47D-KBluc cells were cultured in RPMI-1640 supplemented with 10% FBS (Gibco) at 37 °C in a humified atmosphere of 5% CO_2_ in air.

The studied compounds boscalid, cyprodinil, iprodione, Tamoxifen and estradiol were prepared by dissolving them in DMSO. Compounds were added to the cultured cells for a final concentration in the range of 0.01 µM to 10 µM for fungicides and 0.001nM to 10nM for estradiol and 100nM for Tamoxifen.

### 4.2. Fungicide Cytotoxicity

Fungicide cytotoxicity was studied with the use of an MTT reagent according to the method of Jabłońska-Trypuć et al. [63]. The positive control was cultured with 17β-estradiol, which is a compound commonly used in studies of substances with potential endocrine-disrupting effects in women. Cytotoxicity tests of the analyzed compounds were carried out at concentrations ranging from 10 nmol to 10,000 nmol (10 μM) with the simultaneous use of Tamoxifen (in a concentration of 100 nmol) as a nuclear estrogen receptor (ERα) blocking agent, which allowed us to check whether the analyzed compounds are potential factors causing hormonal disorders and whether they act by binding to ERα in MCF-7 and T47D-KBluc cells [64]. Tamoxifen is an anti-estrogenic drug obtained by chemical synthesis and mainly used in the treatment of breast cancer (ER+). Cells were incubated in a medium without antibiotics and without phenol red, which is an activating factor for the ERα receptor, to exclude its possible interaction with the estrogen receptors of cells, and with the use of estrogen-free serum by treating it with adsorbing agents (carbon active/dialysis).

### 4.3. E-screen Test

The E-screen test was performed on the MCF-7 cell line according to Schiliro T et al. [48]. This bioassay was applied in order to measure an increase in the amount of human MCF-7 breast cancer cells in the presence of estrogenic substances. The MCF-7 cells were seeded in 24-well plates in phenol-red-free medium with filtered serum. After 24 h, the medium was changed to the experimental medium containing dilutions of the tested fungicides. Wells without hormones were negative controls. 17-β-estradiol (E2) at five concentrations (between 1 pM and 10 nM) was the positive control. The one concentration of each fungicide that was found to induce the most proliferation of MCF-7 cells was tested with 5 µM anti-estrogen Tamoxifen and 0.1 nM E2. The assay was terminated after 6 days, and cells were stained with crystal violet to measure absorbance and calculate the relative proliferative effect.

### 4.4. Bioassay for Measurement of Estrogenic Activity

An estrogen receptor (ER) reporter gene assay, referred to as the T47D-KBluc assay, was performed according to methods outlined by Chou et al. [50]. Phenol red and FBS exhibit estrogenic activity, therefore experiments were conducted in RPMI-1640 without phenol red and supplemented with 10% dextran-coated charcoal-treated fetal bovine serum (DCC-FBS) and 1% antibiotics (penicillin/streptomycin). The growth medium was changed to fresh test medium three days prior to the experiment in order to adapt the cells to DCC-FBS. The cells were harvested and seeded in 96-well white plates with flat bottoms at a density of 20,000 cells/well in 100 µL of DCC-FBS per well. After 24 h, the test medium was removed, and 200 µL of medium with the test compounds in different concentrations were added to three wells. The cells were treated for 24 h. A negative control, without hormones, and a positive control, with 17-β-estradiol (E2) (in the concentration range 0.1 to 50 µM), were included in each assay. Luciferase activity was measured using a luminescence plate reader.

### 4.5. Flow Cytometry Detection of Intracellular ROS

The amount of ROS (reactive oxygen species) in the cells was estimated with the use of dichlorodihydrofluorescein diacetate (DCFH-DA), (Sigma, St. Louis, MO, USA) according to protocol in [65]. Cells of both cell lines were seeded in 2 mL of growth medium in 6-well plates. After 24 h, the cells were stained with 10 μM of DCFH-DA in PBS at 37 °C in a 5% CO_2_ incubator for 30 min. Next, the dye was removed and replaced with the studied compounds and incubated for 24 h. Then, the cells were trypsinized, resuspended in medium and then in PBS. The DCF fluorescence intensity was measured by using a FACSCanto II cytometer (Becton Dickinson, Franklin Lakes, NJ, USA). Data were analyzed with FACSDiva software (Ver. 6.1.3. BD Biosciences Systems, San Diego, CA, USA).

### 4.6. Extraction and Preparation of RNA

The total amount of RNA from the MCF-7 and T47DKBluc cells was obtained with the use of the RNeasy Mini Kit (QIAGEN, Hilden, Germany) on QIAcube System (QIAGEN, Hilden, Germany). The cDNA synthesis was performed from 1 µg of each total RNA sample with the use of the iScript cDNA Synthesis Kit (Bio-Rad Laboratories GmbH, Munich, Germany). The transcribed product dilution was about 1:10.0.

### 4.7. Reverse Transcription-Quantitative PCR (RT-qPCR)

Primers for genes encoding glutathione peroxidase (GPx), superoxide dismutase (SOD1) and catalase (CAT) in human cells were obtained from the Bio-Rad collection as PrimePCR ™ SYBR^®^ Green Assay (Bio-Rad Laboratories GmbH, Munich, Germany). GAPDH was used as a reference gene and its sequence and properties have been published by Piana et al. (2008) [66]. All PCRs were performed in 20 µL reaction mixtures containing 1 µL cDNA (diluted 1:10), 10 µL SsoAdvanced™ Universal SYBR^®^ Green Supermix (Bio-Rad Laboratories GmbH, Munich, Germany), 1 µL 20× PrimePCR Assay (for CAT, SOD1, GPx) or 0.5 µL of each reverse and forward primers of GAPDH (10 µM), and 20 µL nuclease-free water. No template controls (NTC) and negative RT samples (reverse transcription omitted) were used for every target gene. Each biological replicate was run in triplicate on a CFX96 Touch Real-Time PCR Detection System (Bio-Rad Laboratories GmbH, Munich, Germany) according to the thermocycling protocol. The results were analyzed using the CFX Manager Software (v3, Bio-Rad Laboratories GmbH, Munich, Germany). Transcript levels were calculated relative to controls and are expressed as the relative normalized expression (2^−ΔΔ*C*t^).

### 4.8. Determination of Surface Charge and Zeta Potential

The surface charge (*δ*) and zeta potential (*ζ*) of MCF-7 and T47D-KBluc cells were determined by conducting microelectrophoretic evaluations of the samples using the electrophoretic light scattering approach. Measurements were conducted with the Zetasizer Nano ZS instrument (Malvern Instruments, Malvern, United Kingdom). The experiment was performed in the pH function via a WTW InoLab pH 720 pH-meter (WTW, Weinheim, Germany).

In brief, samples were resuspended in a 155 mM NaCl solution and titrated to the required pH (2–10 range) using strong acid (HCl) and strong base (NaOH) solutions, which were mixed with NaCl to keep the ionic solution strength fixed. For each pH value, four pH readings (each comprising 100-200 series for a time of 5 s) were taken. The experiment was repeated three times obtaining comparable results.

The (*ζ*) values were obtained from the electrophoretic mobility using the equation [41]:

(1) $$\zeta =\frac{3\cdot \eta \cdot µ}{2\cdot \varepsilon \cdot \varepsilon_{0}\cdot ƒ(\kappa a)}$$

where: *µ*—the electrophoretic mobility, *η*—the viscosity of the aqueous solution, *ε*—the relative permittivity of the medium, *ε*_0_—the permittivity of free space, and *ƒ(κa)*—Henry’s function.

The surface charge values were calculated using the formula [67]:

(2) $$\delta =\frac{\eta \cdot u}{d}$$

in which: *d*—the diffuse layer thickness.

The diffuse layer thickness was determined from the equation [68]:

(3) $$d=\sqrt{\frac{\varepsilon \cdot \varepsilon_{0}\cdot R\cdot T}{2\cdot F^{2}\cdot I}}$$

where: *R*—the gas constant, *T*—the temperature, *F*—the Faraday constant, *I*—the ionic strength of the electrolyte.

### 4.9. LC–ESI–MS/MS Analysis of Boscalid, Cyprodinil and Iprodione

For the LC–ESI–MS/MS analysis, MCF-7 cells were plated in 6-well plates in phenol-red-free medium with filtered serum. After 24 h, the medium was changed to the experimental medium containing the tested fungicides in two analyzed concentrations, 0.01 µM and 0.025µM. Each dilution was tested in 3 replicates for each analysis. After 24 h of incubation, the cells were washed with cold PBS (*w*/*o* Ca and Mg) and they were harvested by scraping in absolute methanol. Subsequently, the cells were transferred for analysis. Analysis was conducted according to the methods of da Costa Morais et al. [69].

### 4.10. Statistical Analysis

In order to estimate every analyzed parameter and each concentration of the fungicides, three to six repetitions were performed. The results obtained are presented as means ± standard deviations (SD) of means for parametric data. Significant differences between the analyzed compounds and their concentrations were estimated by using ANOVA followed by Tukey’s test at *p* ≤ 0.05. Dunnett’s test was applied for comparing the means of data to the control at various significance levels (* *p* < 0.05, ** *p* < 0.01 and *** *p* < 0.001). Scheffe’s F test was applied in order to compare the results of the surface charge (*δ*) and zeta potential (*ζ*) of the MCF-7 and T47DKBluc cells. A * *p* ≤ 0.05 was considered statistically significant. Statistica 13.0 was used.

### 4.11. Morphological Analysis of MCF-7 and T47DKBluc Cells

The cells were stained and analyzed similarly to Krętowski et al. [70] with the use of a fluorescence microscope (Olympus CXK41, U-RLFT50, Microscope Central, Feasterville, PA, USA).

## 5. Conclusions

To sum up, based on the current data, the tested compounds from the group of fungicides, boscalid, cyprodinil and iprodione, clearly show estrogenically active properties. Nevertheless, it is not appropriate to draw conclusions on the potential effects of fungicides on human health on the basis of the in vitro data presented alone. Further research is needed to identify and describe the molecular mechanisms responsible for the action of the selected active substances of fungicides and to assess their impact on human health and the functioning of entire ecosystems. Based on the presented results, it should be concluded that there is a need for the continuous monitoring of these compounds in the environment.

## Figures and Tables

**Table 1:** Physicochemical and toxicological properties of iprodione, boscalid and cyprodinil [16].

Physicochemical/Toxicological Properties	Iprodione	Cyprodinil	Boscalid
**Chemical formula**	C₁₃H₁₃Cl₂N₃O₃	C₁₄H₁₅N₃	C₁₈H₁₂Cl₂N₂O
**2D structure diagram**	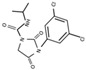	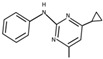	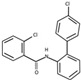
**Molecular mass**	330.17	225.29	343.21
**CAS name**	3-(3,5-dichlorophenyl)-*N*-(1-methylethyl)-2,4-dioxo-1-imidazolidinecarboxamide	4-cyclopropyl-6-methyl-*N*-phenyl-2-pyrimidinamine	2-chloro-*N*-(4′-chloro[1,1′-biphenyl]-2-yl)-3-pyridinecarboxamide
**Solubility—in water at 20 °C** **(mg/L)**	6.8 (Low)	13 (Low)	4.6 (Low)
**Known metabolites**	*N*-(3,5-dichlorophenylcarbamoyl)- *N*-isopropylcarbamoyl-glycine 3,5-dichloroaniline 3-(3,5-dichlorophenyl)-2,4- dioxoimidazolidine 1-[(3,5-dichlorophenyl) carbamoymethyl]-3-isopropylurea	4-cyclopropyl-6-methyl- pyrimidin-2-ol 4-(4-cyclopropyl-6-methyl- pyrimidin-2-yl-amino)-phenol (2-amino-6-cyclopropyl- pyrimidin-4-yl)-methanol 4-(4-cyclopropyl-6-methyl- pyrimidin-2-yl-amino)-phenol	4-chlorobenzoic acid 2-chloronicotinic acid 2-chloro-*N*-(4′-chloro-5-hydroxybiphenyl-2-yl)nicotinamide *N*-(4-chlorobiphenyl-2-yl)-2-hydroxynicotinamide
**Ecotoxicology** **Mammals—acute oral LD₅₀** **(mg/ kg)**	>2000	>2000	>5000
**Human health and protection threshold of toxicological concern (Cramer class)**	High (class III)	High (class III)	High (class III)
**Specific human health issues**	Endocrine disrupter; reproduction/development effects	Mammals’ chronic toxicity: moderate; reproduction/development effects	Possible carcinogen; reproduction/development effects
**ADI—** **Acceptable Daily Intake (mg/kg bw/day)**	0.02	0.03	0.04
**WHO classification**	III (Slightly hazardous)	III (c) (Slightly hazardous/Company classification)	U (Unlikely to present an acute hazard)

**Table 2:** Estrogenic activity of selected fungicides presented by RPE% (relative proliferative effect) in MCF-7 cells and TRANS% (increased rate of luciferase gene expression) in T47D-KBluc gene-reporter luciferase cells.

Fungicides Treatments	E-Screen RPE%	T47DKBluc Luciferase Assay TRANS%
B 0.01 µM	37.212	34.179
B 0.05 µM	42.191	38.028
B 0.1 µM	42.090	41.645
B 0.5 µM	35.885	36.844
B 1 µM	37.544	33.734
B 5 µM	44.161	37.795
B 10 µM	-	95.388
C 0.01 µM	12.590	34.327
C 0.05 µM	10.437	35.744
C 0.1 µM	11.265	43.020
C 0.5 µM	9.026	41.433
C 1 µM	11.923	39.657
C 5 µM	9.259	39.128
C 10 µM	-	95.156
I 0.01 µM	16.358	49.492
I 0.05 µM	16.793	53.172
I 0.1 µM	15.490	72.377
I 0.5 µM	14.575	69.796
I 1 µM	18.919	74.492
I 5 µM	17.889	64.276
I 10 µM	-	83.438

**Table 3:** The comparison of fungicides’ applied doses with the amount of each compound estimated in MCF-7 cells using LC–ESI–MS/MS analysis.

Fungicide	Applied Dose (µg/L)	Amount within the MCF-7 Cells (µg/L)	% Content
Boscalid (0.01 µM)	0.34	0.24	70.5
Boscalid (0.025 µM)	0.85	0.36	42.35
Cyprodinil (0.01 µM)	0.22	0.17	77.27
Cyprodinil (0.025 µM)	0.56	0.22	39.28
Iprodione (0.01 µM)	0.33	0.21	63.63
Iprodione (0.025 µM)	0.825	0.26	31.5

## Data Availability

The data presented in this study are available on request from the corresponding author.
